# A Novel Phage Infecting *Alteromonas* Represents a Distinct Group of Siphophages Infecting Diverse Aquatic Copiotrophs

**DOI:** 10.1128/mSphere.00454-21

**Published:** 2021-06-09

**Authors:** Ruijie Ma, Jiayong Lai, Xiaowei Chen, Long Wang, Yahui Yang, Shuzhen Wei, Nianzhi Jiao, Rui Zhang

**Affiliations:** a State Key Laboratory of Marine Environmental Science, College of Ocean and Earth Sciences, Institute of Marine Microbes and Ecospheres, Xiamen University, Xiamen, China; b College of Ocean and Earth Sciences, Xiamen University, Xiamen, China; c Southern Marine Science and Engineering Guangdong Laboratory (Zhuhai), Zhuhai, China; University of Iowa

**Keywords:** bacteriophage, *Alteromonas*, auxiliary metabolic genes, comparative genomic analysis, phage taxonomy, receptor binding protein

## Abstract

Bacteriophages play critical roles in impacting microbial community succession both ecologically and evolutionarily. Although the majority of phage genetic diversity has been increasingly unveiled, phages infecting members of the ecologically important genus *Alteromonas* remain poorly understood. Here, we present a comprehensive analysis of a newly isolated alterophage, vB_AcoS-R7M (R7M), to characterize its life cycle traits, genomic features, and putative evolutionary origin. R7M harbors abundant genes identified as host-like auxiliary metabolic genes facilitating viral propagation. Genomic analysis suggested that R7M is distinct from currently known alterophages. Interestingly, R7M was found to share a set of similar characteristics with a number of siphophages infecting diverse aquatic opportunistic copiotrophs. We therefore proposed the creation of one new subfamily (*Queuovirinae*) to group with these evolutionarily related phages. Notably, tail genes were less likely to be shared among them, and baseplate-related genes varied the most. In-depth analyses indicated that R7M has replaced its distal tail with a Rhodobacter capsulatus gene transfer agent (RcGTA)-like baseplate and further acquired a putative receptor interaction site targeting *Alteromonas*. These findings suggest that horizontal exchanges of viral tail adsorption apparatuses are widespread and vital for phages to hunt new hosts and to adapt to new niches.

**IMPORTANCE** The evolution and ecology of phages infecting members of *Alteromonas*, a marine opportunistic genus that is widely distributed and of great ecological significance, remain poorly understood. The present study integrates physiological and genomic evidence to characterize the properties and putative phage-host interactions of a newly isolated *Alteromonas* phage, vB_AcoS-R7M (R7M). A taxonomic study reveals close evolutionary relationships among R7M and a number of siphophages infecting various aquatic copiotrophs. Their similar head morphology and overall genetic framework suggest their putative common ancestry and the grouping of a new viral subfamily. However, their major difference lies in the viral tail adsorption apparatuses and the horizontal exchanges of which possibly account for variations in host specificity. These findings outline an evolutionary scenario for the emergence of diverse viral lineages of a shared genetic pool and give insights into the genetics and ecology of viral host jumps.

## INTRODUCTION

Viruses have been acknowledged as the most abundant life forms in marine ecosystems. Generally, viral abundance exceeds prokaryotic abundance by a factor of 10 in the upper ocean (1). Despite being extremely small, viruses can exert critical influences by shaping microbial communities, promoting biogeochemical cycling of nutrients, and driving the evolution of microbial hosts (2). Furthermore, viruses exhibit high recombination rates and frequently exchange their genetic components with hosts and other viruses, representing the largest reservoir of genetic diversity (3). Culture-independent technological advances, such as viral high-throughput sequencing and metagenomic analysis of bulk viral community, have improved our knowledge of viral diversity and virus-host interactions. However, to continually interpret viral dark matters from environmental samples and to unveil novel information in specific virus-host systems, culture-dependent methods are still of great significance.

The genus *Alteromonas* represents a group of marine gammaproteobacterial copiotrophs (actively blooming microbes) that have a worldwide distribution (4). At the time of writing, the genus *Alteromonas* comprises a total of 28 species with validly published names (https://www.bacterio.net/genus/alteromonas). Previous studies have shown that, as typical opportunistic copiotrophs, members of *Alteromonas* can utilize largely diverse carbon sources and rapidly degrade labile organic matter in the aquatic environment (5, 6), implying their crucial roles in global carbon and nutrient cycling. Moreover, *Alteromonas* acts as a helper bacterium that shields *Prochlorococcus*, an important marine primary producer in the oligotrophic regions, from being damaged by hydrogen peroxide produced during photochemical reactions (7). As one of the most important bacteria in marine ecosystems, *Alteromonas* has received increasing attention and gradually become a model organism.

Compared with the improved understanding of *Alteromonas* in the past decade, much less is known about its phages. To date, only 12 complete sequences of alterophages, isolated using hosts from 7 *Alteromonas* species, have been deposited in the GenBank database (8–13) (Table 1). Not all of these alterophages have been characterized and described in depth, which hinders us from further understanding the alterophage diversity, phage-*Alteromonas* interactions, and viral influences on *Alteromonas* genetic evolution. Here, we report a newly isolated phage, vB_AcoS-R7M, infecting Alteromonas confluentis using DSSK2-12^T^ (14) as a host. The present study combined physiological and genomic evidence to reveal the properties and functional interpretation of alterophages and their roles in *Alteromonas* ecology. The finding of evolutionary relevance among phages infecting diverse aquatic copiotrophs promoted the creation of a new viral subfamily and had broader implications for viral evolution, host specificity, and ecological niche specialization.

**TABLE 1:** Detailed information on the currently known *Alteromonas* phages

Phage	Host	Isolation source	Morphology	Length (bp)	G+C (%)	GenBank accession no.	Reference
vB_AmaP_AD45-P1	Alteromonas macleodii AD45	Coastal water, Altea, Spain	*Podoviridae*	103,910	43.2	KF005317.1	8
vB_AmaP_AD45-P2	*A. macleodii* AD45	Coastal water, Altea, Spain	*Podoviridae*	104,036	43.2	KF005320.1	8
vB_AmaP_AD45-P3	*A. macleodii* AD45	Coastal water, Altea, Spain	*Podoviridae*	101,724	43.2	KF005318.1	8
vB_AmaP_AD45-P4	*A. macleodii* AD45	Coastal water, Altea, Spain	*Podoviridae*	100,619	43.2	KF005319.1	8
PB15	Alteromonas gracilis B15	Yellow Sea, China	*Siphoviridae*	37,333	45.5	KX982260.1	9
vB_AspP-H4/4	*A. addita* H4	North Sea water near Helgoland, Germany	*Podoviridae*	47,631	40.8	MF278336.1	10
JH01	*A. marina* SW-47^T^	Qingdao coast, China	*Siphoviridae*	46,500	44.4	MH445500.1	11
vB_AmeM_PT11-V22	Alteromonas mediterranea PT11	Surface seawater, Spain	*Myoviridae*	92,760	38.4	MN877442.1	12
P24	*A. macleodii*	Qingdao coast, China	*Siphoviridae*	33,567	43.7	MK241539.2	13
ZP6	*A. macleodii*	China	*Podoviridae*	37,743	50.1	MK203850.1	Unpublished
XX1924	Alteromonas litorea TF-22^T^	Yellow Sea, China	*Siphoviridae*	40,580	43.7	MN592896.1	Unpublished
vB_AcoS-R7M	*A. confluentis* DSSK2-12^T^	Xiamen coast, China	*Siphoviridae*	56,163	45.6	MT345684.1	This study

## RESULTS AND DISCUSSION

### Morphology and biological features of R7M.

**FIG 1:**
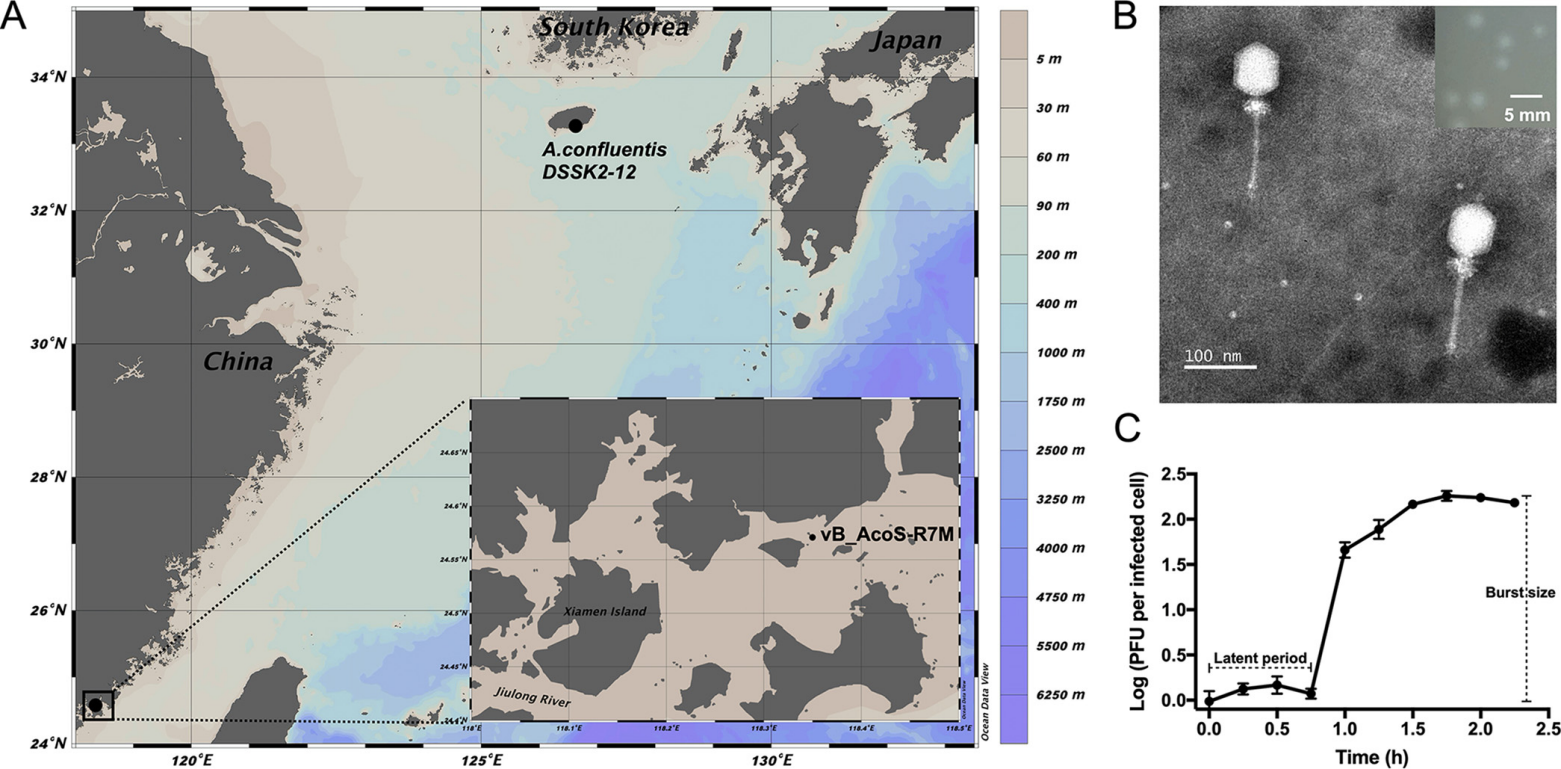
Isolation and biological features of R7M. (A) Map showing the phage sampling site and the host origin, generated using Ocean Data View (version 4.7.10; Schlitzer, R., Ocean Data View, https://odv.awi.de/, 2017). (B) Transmission electron micrograph of R7M. Scale bar, 100 nm. Inset shows plaques of R7M formed on the lawn of *A. confluentis* DSSK2-12^T^ with a scale bar of 5 mm. (C) One-step growth curve of R7M. Each data point is shown as the mean ± SD of three independent replicates, and the figure was drawn using GraphPad Prism 7 (GraphPad, CA, USA).

Using Alteromonas confluentis DSSK2-12^T^ as the host that was isolated from nearshore water (33°15′7″N, 126°37′26″E) near Jeju Island of South Korea (14), we isolated an alterophage named R7M from coastal eutrophic surface water (24°34′20″N, 118°21′16″E) near Dadeng Island, Xiamen, China (Fig. 1A). The plaque size ranges from 1.5 to 2.0 mm after 24 h. Clear plaques are nearly round with a turbid halo around them (Fig. 1B), indicative of the presence of viral soluble enzymes that degrade extracellular polymeric substances of mucoid host cells (15). Phage R7M exhibits *Siphoviridae* morphology with an elongated head (72.65 ± 1.34 [mean ± SD] nm long and 59.25 ± 1.19 nm wide) and a long flexible tail (137.51 ± 2.62 nm long). The transmission electron microscopy (TEM) revealed the presence of hairy fiber-like whiskers at the phage head-tail interface (Fig. 1B), which were found in alterophages for the first time. Previous studies reported the presence of the collar/whisker complex in Escherichia coli bacteriophage T4 and in a few lactococcal siphophages (16, 17). T4 whiskers were demonstrated to play a role as an environmental sensor that controls extension and retraction of phage tail fibers (16), while those in lactococcal siphophages do not affect either phage assembly or host range (17). Noticeably, R7M vastly exceeds the number of whiskers compared with those mentioned above, and thus, the function of whiskers in R7M awaits further study.

To explore the life cycle of R7M, a one-step growth curve was examined in this study. Our results showed that R7M was more likely to follow a lytic life strategy featuring a short latent period (45 min) and a rapid growing period (60 min). Overall, it took less than 2 h for R7M to fulfill one round of propagation (Fig. 1C). Phage R7M exhibits the largest burst size of approximately 182 PFU/cell compared with other currently known siphophages infecting *Alteromonas* (60 to 147 PFU/cell) (8, 9), showing a strong lytic ability.

**FIG 2:**
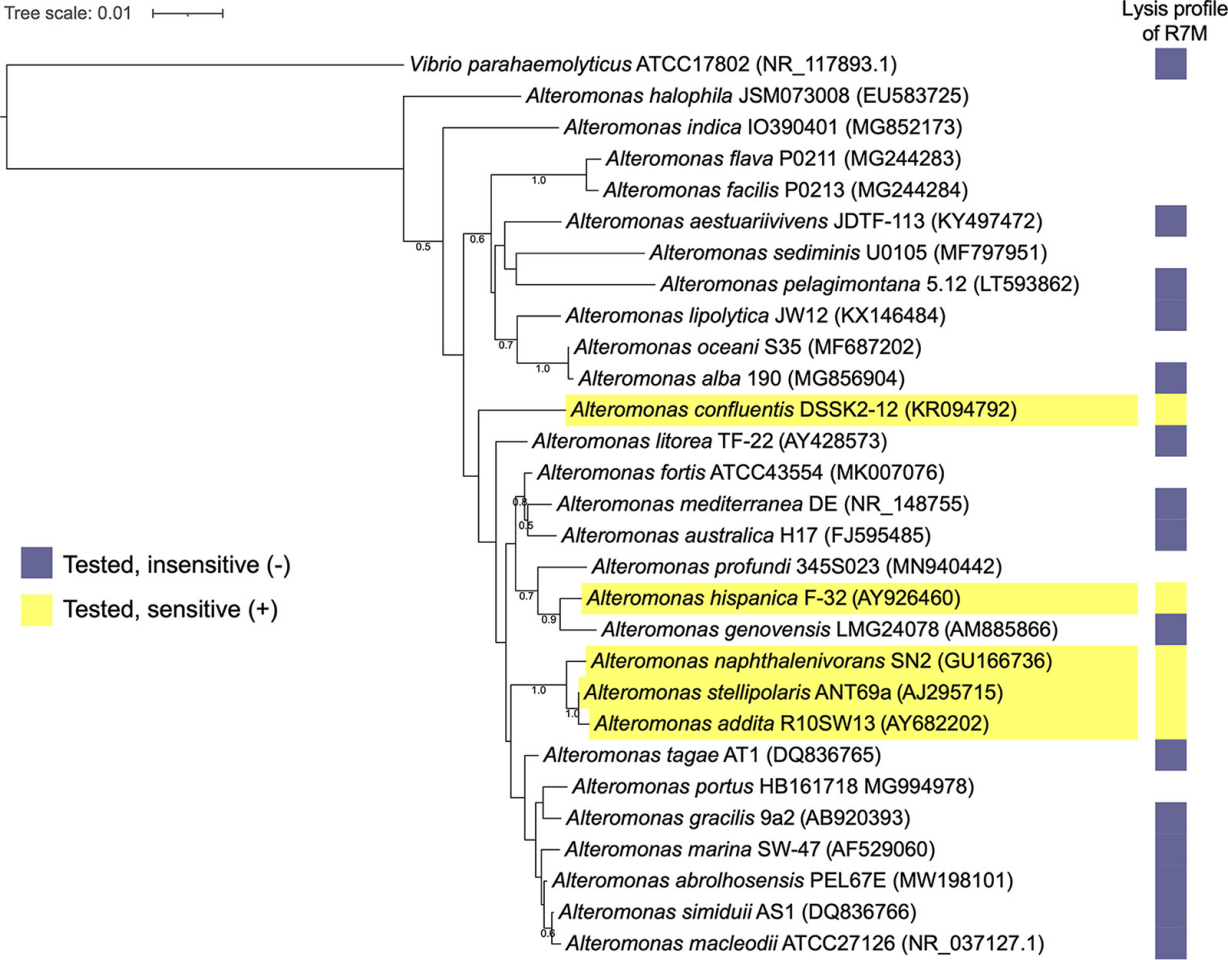
R7M lysis profiles of different type strains of species of the *Alteromonas* genus. Sensitive hosts are highlighted using yellow shadows.

A cross-infectivity test was performed to examine the host range of R7M within an extensive collection of 18 type strains of species of the genus *Alteromonas*. Such a large survey covers more than one-half of the currently known *Alteromonas* type strains (18 out of 28). Three *Vibrio* spp., belonging to *Gammaproteobacteria* class, were included as well. Our results showed that R7M cannot lyse 3 *Vibrio* spp. tested, but of 18 *Alteromonas* type strains tested, R7M can lyse 5 of them (Fig. 2). Except for the original host, R7M could also lyse Alteromonas hispanica F-32^T^, Alteromonas naphthalenivorans SN2^T^, Alteromonas stellipolaris ANT69a^T^, and Alteromonas addita R10SW13^T^. The latter two type strains, sharing 99.59% 16S rRNA gene sequence identity and 98.93% average nucleotide identity (18), could be merged into a single species. Thus, R7M is able to lyse type strains of at least four different species of the genus *Alteromonas*, covering a relatively wide host range. Susceptible strains comprised three isolated from coastal waters (DSSK2-12^T^, SN2^T^, and R10SW13^T^), one from inland hypersaline water (F-32^T^), and one from the Antarctic Ocean (ANT69a^T^) (see Table S1 in the supplemental material).

10.1128/mSphere.00454-21.5 TABLE S1 Strains used in the host-range test and their susceptibility to R7M. Download Table S1, PDF file, 0.07 MB. Copyright © 2021 Ma et al. 2021 Ma et al. https://creativecommons.org/licenses/by/4.0/ This content is distributed under the terms of the Creative Commons Attribution 4.0 International license.

### Overview of genomic features of R7M.

**FIG 3:**
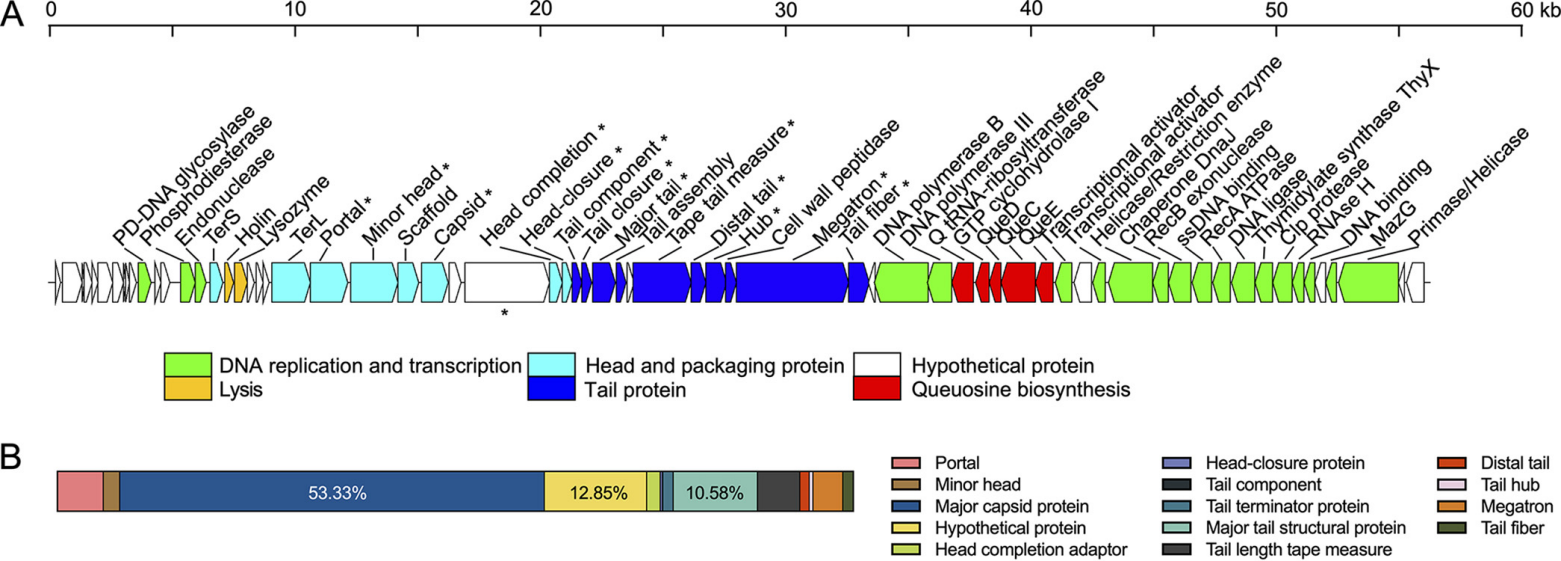
(A) The genome map of R7M. The orientation of each ORF corresponds to the direction of transcription. Genes within different functional categories are indicated by colors noted below. (B) Relative abundance of R7M structural proteins detected in ESI-MS/MS.

Sequencing resulted in a total of 14,136,773 clean reads and 1 complete contig with a length of 56,163 bp and an average sequencing coverage of 1,763×. Phage R7M has a circular double-stranded DNA genome with a G+C content of 45.61%, which is lower than that of its host (48.02%). No tRNA genes were identified in R7M, and no lysogeny-related hallmarks (transposase or integrase, excisionase, and repressor) were detected in R7M. Along with the rapid burst in reproduction, we therefore infer that R7M is a lytic phage. In total, 67 putative open reading frames (ORFs) were predicted in the phage genome, which was tightly packed and presented a coding density of 95.28%. Similar to most phages, the R7M genome exhibited an overall modular organization (Fig. 3A). Among all the ORFs, 44 (65.67%) were annotated as functional proteins, while the others were assigned as hypothetical proteins with unknown functions. To expand our understanding of genes encoding the R7M structure, mass spectrometry-based structural proteomics was conducted. A total of 14 structural proteins were detected in purified virus particles (Fig. 3B; see also ORFs denoted as asterisks in Fig. 3A), including 1 hypothetical protein and 13 proteins with predicted function as structural proteins. An overview of mass spectrometry data for R7M was deposited in Table S2 in the supplemental material. Of note, hypothetical protein ORF28 reached a second-highest coverage (12.85%) only next to the phage major capsid protein (53.33%) and was followed by the phage major tube protein (10.58%) (Fig. 3B). Such a high ratio implies that ORF28 is an essential participant in R7M virion construction. Given that ORF28 was present in a head-tail module, it is possible that ORF28 encodes R7M whiskers.

10.1128/mSphere.00454-21.6 TABLE S2 Mass spectrometry data for vB_AcoS-R7M. A minimal of two unique peptides and 5% sequence coverage were used as threshold values. Download Table S2, PDF file, 0.1 MB. Copyright © 2021 Ma et al. 2021 Ma et al. https://creativecommons.org/licenses/by/4.0/ This content is distributed under the terms of the Creative Commons Attribution 4.0 International license.

### Phage-host interactions inferred from R7M genes.

**Pyrimidine dimer DNA glycosylase (PDG).** As common UV-induced photoproducts, pyrimidine dimers are one of the major DNA lesions in both microbial and phage genomes, leading to cell death and phage inactivation (19). It was shown that mutations in the bacteriophage T4 *denV*, which encodes T4 PDG and functions to remove pyrimidine dimers, resulted in the increased sensitivity to UV radiation (20). The T4 *denV* and other *denV*-like genes cloned in host cells complemented host deficiency in UV resistance (21, 22). In the R7M genome, ORF11 was predicted to be PDG. Despite lacking homology at the sequence level compared with T4 PDG, ORF11 was structurally related to T4 PDG (TM score, 0.795) (see Fig. S1 in the supplemental material), which strongly supports that ORF11 and T4 PDG should have similar functions and a common precursor since protein sequences usually diverge earlier than their structures (23).

10.1128/mSphere.00454-21.1 FIG S1 Structural comparison of R7M PDG and T4 PDG. (A) The 3D predicted structure of R7M PDG (ORF11). (B) Crystal structure of T4 PDG (PDB ID: 1ENK; K. Morikawa, M. Ariyoshi, D. G. Vassylyev, O. Matsumoto, K. Katayanagi and E. Ohtsuka, J Mol Biol 249:360-375, 1995, doi:https://doi.org/10.1006/jmbi.1995.0302). (C) Structure superposition of these two enzymes (TM-score, 0.795), with R7M PDG shown in cartoon (rainbow) and T4 PDG displayed using backbone trace (purple). The shared domain PF03013 is outlined using a black dashed line. Download FIG S1, PDF file, 0.2 MB. Copyright © 2021 Ma et al. 2021 Ma et al. https://creativecommons.org/licenses/by/4.0/ This content is distributed under the terms of the Creative Commons Attribution 4.0 International license.

This is the first example of an alterophage encoding such a host-independent UV repair system. In general, viral DNA repair systems are of vital importance to the survival and persistence of virions in the ocean, especially in upper waters characterized by strong UV radiation. Such phages can restore their activities with the aid of host photoreactivating enzymes (photolyase) in the light (24), or they can remove the damaged section by self-encoded repair genes in the dark, followed by the sealing of DNA nicks via DNA polymerase I (ORF43 in R7M) and DNA ligase (ORF58 in R7M).

**Thymidylate synthase ThyX.** Thymidylate synthase is widely involved in the *de novo* synthesis of dTMP using dUMP as a substrate. Genes for this enzyme include *thyA* (the canonical form) and *thyX* (the alternative form), with *thyX* involved in a simpler pathway (25). In R7M, ORF59 was predicted to be FAD-dependent ThyX (Pfam PF02511.10), which is present in approximately one-half of all known alterophages (8, 10, 12). Viral *thy* has been assumed to be involved in nucleotide salvage, enabling viruses to scavenge host nucleotides and synthesize thymidylate, which is even more important when host transcription terminates (26). As potential vehicles in horizontal gene transfer (HGT), phages have been proposed to promote *thy* transport among bacterial hosts (25, 27). All *Alteromonas* genomes in the GenBank contain *thyA*, while only three of them also carry *thyX* (Refseq accession WP_136782054.1, MAI39418.1, and WP_119501142.1). Further analysis indicated that the three *Alteromonas* genomes acquired their *thyX* horizontally with the aid of mobile genetic elements. For example, the *thyX* in *Alteromonas* sp. RKMC-009 (WP_119501142.1) was found in a full-length N4-like prophage. This finding highlights a possibility that *thyX*-carrying alterophages (or other mobile genetic elements) may provide alternative complementary pathways of nucleotide metabolism for their hosts through HGT. Thus, we propose that viral *thyX* should be considered a new member of class II auxiliary metabolic genes (AMGs) that play a more peripheral role in host metabolism.

**Nucleotide pyrophosphohydrolase MazG.** MazG in Escherichia coli enables the cell to cease the programmed cell death by hydrolyzing guanosine tetraphosphate (p)ppGpp, which is generated in response to amino acid starvation, thereby enhancing host survival under nutrient-depleted environments (28). A similar role has been proposed for virus-encoded MazGs (29), and therefore, it has long been held that *mazG*-carrying phages may modulate the metabolism of host cells during infection to ensure a sufficient proliferation of progeny virions (30). Such a role in host stringer responses enables MazG to be grouped into class I AMGs (31). However, a recent study suggested that an investigated cyanophage-encoded MazG showed no binding or hydrolysis ability against (p)ppGpp. Instead, a preference for dGTP and dCTP as substrates was observed, suggesting a role in recycling host nucleotides (32). In this study, a MazG-like domain (Pfam PF03819.12) was found within ORF64 of phage R7M, and it is not clear whether a similar scavenging role exists in R7M ORF64. Nevertheless, more and more findings of MazG in viruses infecting hosts isolated from nutrient-rich environments (33, 34) indicate the need for examining the enzymatic activity of phage-encoded MazGs from more diverse sources (e.g., marine/terrestrial, autotrophic/heterotropic, or oligotropic/copiotropic).

**Queuosine biosynthesis genes.** Genome modifications are of great importance in the phage-host evolutionary arms race. 7-Deazaguanine modifications, previously discovered in tRNA as queuosine (Q) in bacteria and archaeosine (G+) in archaea, were recently found in bacterial and viral DNA (35). 7-Cyano-7-deazaguanine (preQ_0_), a key intermediate in both the Q and G+ pathways, can be synthesized from GTP by four enzymes (FolE, QueD, QueE, and QueC). Some viruses encoded DpdA/DpdA2 and Gat-queC associated with 2’-deoxy-archaeosine (dG+) synthesis using preQ_0_ as a substrate (35). Thiaville et al. revealed that about 25% to 27% of the 2′-deoxy-guanosine (dG) in E. coli phage 9g (*Nonagvirus*) DNA was replaced by dG+ (36). A subsequent *in vitro* experiment verified that dG+ modifications rendered phage 9g DNA resistant to about 71% of type II restrictions (37). In R7M, such an entire dG+ synthesis pathway was identified (ORF45 to ORF49: DpdA, FolE, QueD, Gat-queC, and QueE, respectively) and proved to be close homologs of those in phage 9g (see Fig. S2 in the supplemental material). R7M also encodes a set of enzymes necessary to incorporate dG+ (e.g., DNA primase, ligase, and polymerase). As a result, R7M was predicted to modify its DNA with dG+ to protect phage DNA from host restriction systems, as does phage 9g. This likely contributes to the wide host range of R7M in different *Alteromonas* species.

10.1128/mSphere.00454-21.2 FIG S2 Queuosine biosynthesis genes in R7M. (A) Genomic context of genes for dG+/preQ_0_ pathways in phage representatives of the subfamily *Queuovirinae*. Genes with different functions are indicated by colors noted in the legend. (B) Phylogenetic analysis based on alignment of the aligned sequence among the Gat-QueC and QueC domains. Trees were rooted in the midpoint, and branches were colored accordingly. Download FIG S2, PDF file, 0.2 MB. Copyright © 2021 Ma et al. 2021 Ma et al. https://creativecommons.org/licenses/by/4.0/ This content is distributed under the terms of the Creative Commons Attribution 4.0 International license.

It is noteworthy that, although no viruses carry QueAGH (only found in bacteria) for Q synthesis, phages could increase the level of Q in host tRNAs with the production of Q precursors (35) and thus improve host translational efficiency (38). Global protein translation could be regulated by viruses containing genes involved in the Q synthesis pathway. Thus, viral-encoded queuosine biosynthesis genes were categorized as class II AMGs (31).

### The RcGTA-like baseplate with a mosaic tail fiber.

**FIG 4:**
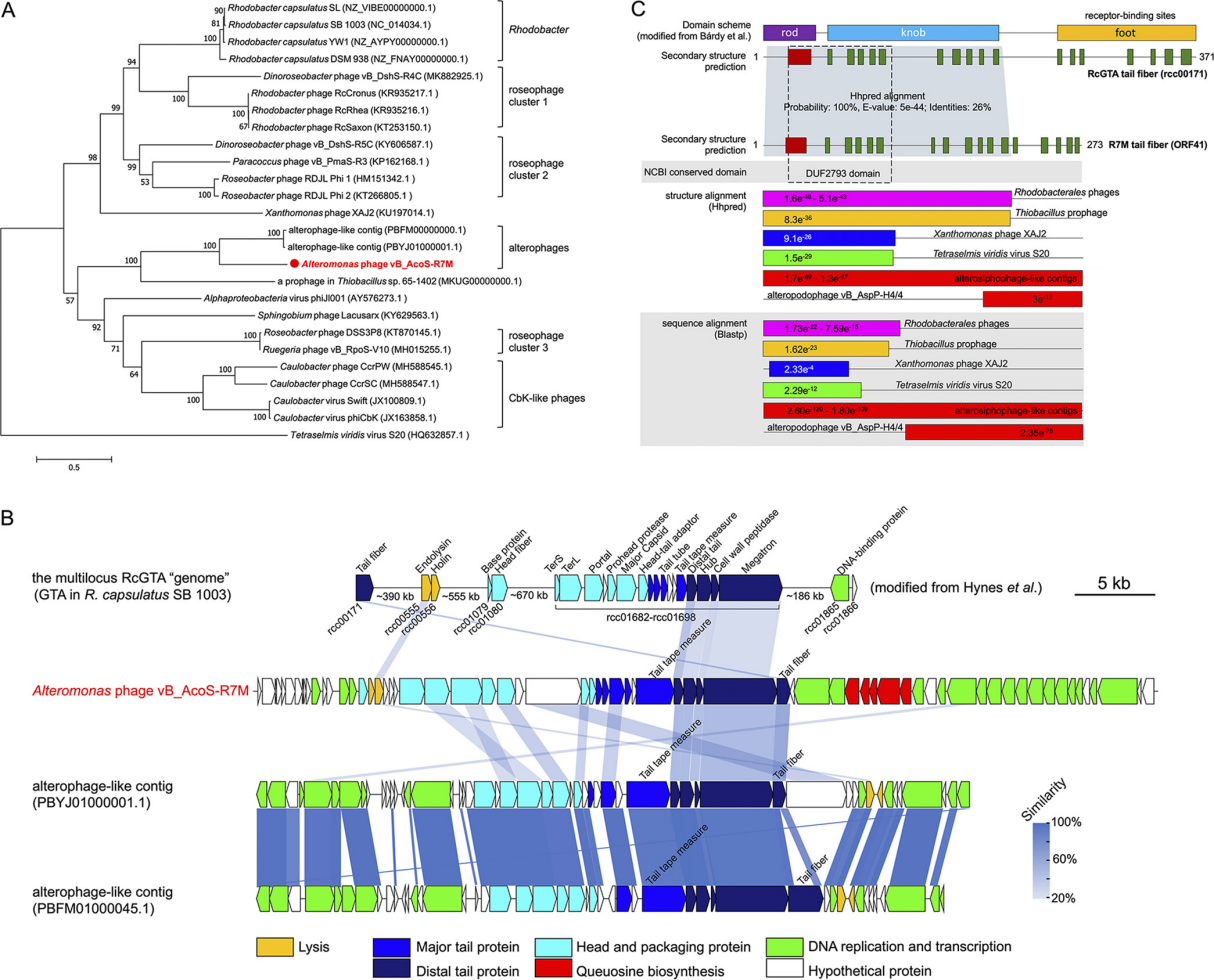
The RcGTA-like baseplate in R7M. (A) The maximum-likelihood phylogenetic tree based on concatenated translated sequences of the four hallmark RcGTA-like genes from R7M and other known viruses or bacteria. Bootstrap values were based on 1,000 replicates. R7M is highlighted in bold with red color. (B) Full-genome comparison of the multilocus RcGTA “genome” (40) and members of the new alterophage clade. Homologous proteins are indicated and connected using blue shadings. The difference in the transparency of shadings corresponds to the similarity between two genes. (C) Secondary structure alignment and amino acid sequence alignment against tail fibers from RcGTA and other tailed viruses. Predicted helices are marked as red columns, and sheets are marked as green columns. E values of HHpred and BLASTP alignments are shown in the figure. Three domains (rod, knob, and foot) in RcGTA tail fiber (rcc00171) were previously identified by Bárdy et al. (42).

GTA was first discovered in the purple photosynthetic alphaproteobacterium Rhodobacter capsulatus and considered the remnant of a phage ancestor (39, 40). It can form phage-like particles (RcGTA) that mediate HGT between donor and receptor cells through transduction. RcGTA-like genes could also be found in numerous viral genomes; however, only baseplate-related genes (rcc01695 to rcc01698: distal tail, tail hub, cell wall peptidase, and megatron, respectively) were mostly preserved (e.g., ORF37 to ORF40 in R7M). Previously, RcGTA-like baseplates were mainly found in marine roseophages and phiCbK-like phages infecting aquatic *Caulobacter*. Our study discovers the presence of RcGTA-like baseplates for the first time in phages infecting *Alteromonas*. A phylogenetic analysis based on these four hallmark RcGTA-like genes indicated that R7M forms a new clade with two viral contigs found in metagenome-assembled genomes recruited from the Pacific Ocean (41) (Fig. 4A and B). These two contigs share ∼95% average nucleotide identity with some of our unpublished lytic alterosiphophages (data not shown). Here, we use “altero(sipho)phage-like contigs” to refer to these two contigs. Such a distinct alterophage clade indicated that a long-term independent evolution of RcGTA-like baseplates existed in alterophages.

A recent cryo-electron microscopy (cryo-EM) reconstruction of RcGTA particles showed that the RcGTA baseplate consists of rcc01695 to rcc01698 and a tail fiber (rcc00171), which contains the receptor-binding site for host recognition and adsorption (42). Interestingly, the N terminus of R7M ORF41 shares a conserved DUF2793 domain (Pfam PF10983) with that of rcc00171, while two genes vary greatly with their C termini, on which the receptor-binding sites are based (Fig. 4C). A structural comparison of rcc00171 and ORF41 using HHpred (43) and TM-align (44) further confirmed this result (Fig. 4C; see also Fig. S3 in the supplemental material). In contrast, the C terminus of ORF41 matches well with those of tail fibers from the alteropodophage vB_AspP-H4/4 (10) and two alterosiphophage-like contigs both genetically and structurally, whereas it lacks hits with viruses from other hosts. This finding indicates that ORF41 is a putative receptor-binding protein (RBP) specific to *Alteromonas* hosts. Thus, we speculate that the two RBPs were derived from common ancestry as they need conserved N termini to ensure a right attachment to the megatron fiber-binding domain (42), while their heterogeneous C termini are to fit with specific surface receptors of its coevolved hosts.

10.1128/mSphere.00454-21.3 FIG S3 Structural comparison of R7M tail fiber (ORF41) and RcGTA tail fiber (rcc00171). (A) 3D prediction of R7M tail fiber. (B) 3D prediction of RcGTA tail fiber. The N termini and C termini are indicated with blue and red, respectively. (C) Structure superposition of these two tail fibers (RBPs) (TM-score, 0.209). Download FIG S3, PDF file, 0.7 MB. Copyright © 2021 Ma et al. 2021 Ma et al. https://creativecommons.org/licenses/by/4.0/ This content is distributed under the terms of the Creative Commons Attribution 4.0 International license.

### R7M inspired the creation of a new subfamily, *Queuovirinae*, of *Siphoviridae*.

**FIG 5:**
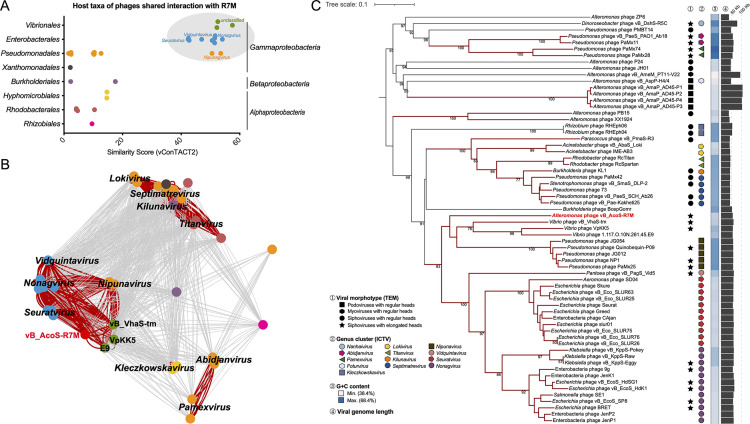
Evidence supporting the taxonomic standing of R7M. (A) Overview of pairwise similarity scores among R7M and its related phages determined by vConTACT2, with a cutoff of >1. (B) Protein-sharing network indicating evolutionary affinity among R7M and its related phages sharing pairwise similarity scores of >1. Each node represents a phage genome and is colored according to its host taxonomy. In particular, edges connecting pairwise phages from the same viral cluster determined by vConTACT2 are displayed in bold and colored in red. The valid names of existing phage genera are displayed on the figure. (C) The GBDP tree based on complete amino acid profiles of compared phages. The numbers above branches are GBDP pseudobootstrap support values from 100 replications. Clades containing phages from the same viral cluster are colored in red. Viral morphotypes are marked according to their published TEM pictures, and the genus cluster information was obtained from the ICTV.

To explore the taxonomic standing of R7M, vConTACT2 v0.9.19 (45) was used to compare R7M gene contents against the ProkaryoticViralRefSeq99 (v99) database. A total of 35 *Proteobacteria* phage representatives showing a similarity score of >1 was detected (Data set S1 in the supplemental material). R7M showed high scores (>40) compared with *Gammaproteobacteria* phages from the genera *Nipunavirus*, *Nonagvirus*, *Seuratvirus*, and *Vidquintavirus*, as well as a few unassigned *Vibrio* phages (Fig. 5A), and these phages also formed a distinct viral cluster defined by vConTACT2 (Data set S1 in the supplemental material). Most of these *Gammaproteobacteria* phages were isolated using hosts such as *Vibrio*, Pseudomonas, and enterobacteria. Similar to *Alteromonas*, they are opportunistic copiotrophs that grow fast and often dominate nutrient-rich environments. Such a grouping was also verified by the gene content-based network employing an edge-weighted spring-embedded layout, in which R7M lies more closely together with phages of the same viral cluster (Fig. 5B). Considering only certain phage representatives of each genus were included in the v99 database, we provided a most up-to-date list with a detailed description of members of these affiliated genera in Data set S2 in the supplemental material.

10.1128/mSphere.00454-21.7 DATA SET S1 A list of viral genomes in each viral cluster in the network analysis. Download Data Set S1, XLSX file, 0.01 MB. Copyright © 2021 Ma et al. 2021 Ma et al. https://creativecommons.org/licenses/by/4.0/ This content is distributed under the terms of the Creative Commons Attribution 4.0 International license.

10.1128/mSphere.00454-21.8 DATA SET S2 Detailed information on phages of the newly proposed subfamily *Queuovirinae*. Download Data Set S2, XLSX file, 0.02 MB. Copyright © 2021 Ma et al. 2021 Ma et al. https://creativecommons.org/licenses/by/4.0/ This content is distributed under the terms of the Creative Commons Attribution 4.0 International license.

To further test whether R7M and any alterophage could be related, we also constructed a phylogenetic tree based on pairwise comparisons of amino acid sequences of R7M, all known alterophages, and a full collection of related phages detected by vConTACT2. In the resulting tree, R7M failed to form reliable clustering with any known alterophage, which again reflects a significant scarcity of reported alterophage isolates. Instead, the clustering of R7M and related *Gammaproteobacteria* phages showed a reliable genome BLAST distance phylogeny (GBDP) pseudobootstrap support value (83 in 100 replications) (Fig. 5C). Of note, all those grouped phages, including R7M, belong to *Siphoviridae*, with an elongated head (morphotype B2 [46]; asterisks marked in Fig. 5C) as evidenced by their published TEM images. Phylogenetic trees constructed using viral hallmarks, including the major capsid protein, terminase large subunit, and DNA polymerase B (Fig. S4 in the supplemental material), also support their close evolutionary relationships.

10.1128/mSphere.00454-21.4 FIG S4 Phylogenetic trees constructed using viral hallmarks, including the major capsid protein (A), terminase large subunit (B), and DNA polymerase B (C). Trees were rooted in the midpoint, and members in the subfamily *Queuovirinae* were colored according to their host taxa as for Fig. 5A. Valid names of existing phage genera are indicated. Download FIG S4, PDF file, 0.2 MB. Copyright © 2021 Ma et al. 2021 Ma et al. https://creativecommons.org/licenses/by/4.0/ This content is distributed under the terms of the Creative Commons Attribution 4.0 International license.

Based on a well-recognized criterion for the classification of viruses (47), namely, that members within the same genus should share >50% pairwise nucleotide sequence similarity across their whole-genome lengths, we realized that R7M should be categorized into a new genus as a type phage since it only shared <2% whole genome with *Vibrio* phage vB_VhaS-tm (GenBank KX198614.1) determined by BLASTN (Data set S2 in the supplemental material). Due to the overall novelty of the R7M nucleotide sequence, we therefore propose a new bacteriophage genus, *Amoyvirus*, under the family of *Siphoviridae* of the *Caudovirales* order, with R7M as the type phage (ICTV assigned code: 2020.128B). Also, because the genus *Amoyvirus* and the other four existing genera (*Nipunavirus*, *Vidquintavirus*, *Nonagvirus* and *Seuratvirus*) shared a set of morphological and genetic characteristics, we further grouped them into a new subfamily, *Queuovirinae* (ICTV assigned code: 2020.128B), which is a name inspired by the presence of *que* genes found in all members of this subfamily.

### Horizontal exchanges of viral distal tail genes lead to differential host specificity.

**FIG 6:**
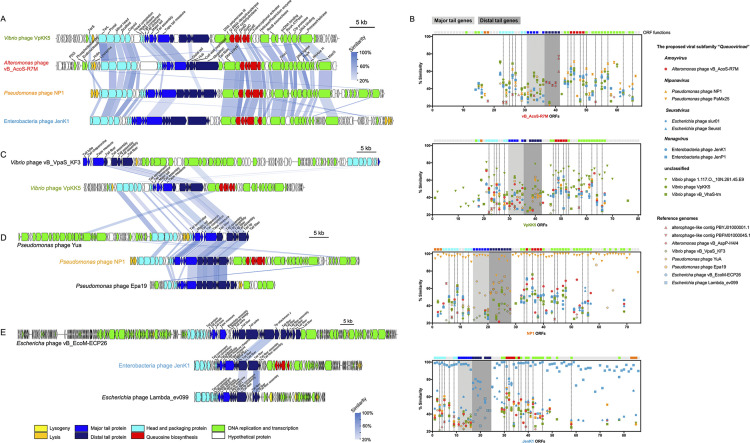
Comparative genomic analyses revealing the evolutionary origin of R7M. (A) Full-genome comparison of the four phage representatives from the subfamily *Queuovirinae*. (B) ORF homologs of the four phage representatives searching against a data set composed of *Queuovirinae* genomes and reference viral genomes. Core genes that are present in all members of *Queuovirinae* are indicated with dash lines. Here, phage tail genes are divided into two categories, namely, genes for the major tail (light gray shadows) and the distal tail (dark gray shadows), respectively. (C to E) Putative horizontal transfers of distal tail genes among unrelated phages infecting *Vibrio* (C), Pseudomonas (D), and enterobacteria (E). Notations and shadings are as for Fig. 4B.

To further explore the evolutionary origin of R7M, representatives within the *Queuovirinae* subfamily, including *Vibrio* phage VpKK5 (GenBank KM378617.2), Pseudomonas phage NP1 (KX129925.1), and enterobacterium phage JenK1 (KP719134.1), were chosen to compare to R7M in depth (Fig. 6A). Although these affiliated phages are almost completely irrelevant at the nucleotide level, they are highly related at the protein level and exhibit a high degree of consistency in their overall genomic architectures, which implies that these phages have diverged from their last common ancestor for a very long time. Conserved fragments occupy most of the genome of these viruses. Lysis-related and tail genes, however, are less likely to be shared, which is in line with our expectations that viruses are likely to expand their habitats through the acquisition of new tail adsorption devices to hunt new hosts and optimize their infection-related apparatuses to better adapt to the desired targets.

Here, we further classified phage tail-related genes into two categories, namely, the major tail (proximal to the capsid) and the distal tail (opposite to the capsid). The major tail contains genes for the tail terminator, major tube, and tape measure, while the distal tail includes genes for the tail baseplate (hub, tail fiber, and tail tip) and baseplate assembly. Interestingly, homologs of major tail genes are relatively more conserved within the subfamily members, whereas homologs of distal tail genes are often present in phages targeting hosts of the same taxon, as seen in R7M and enterobacterium phage JenK1 (Fig. 6B). Evidence also revealed that several unrelated phages targeting the same host are similar in their distal tail genes (Fig. 4B and 6C to E). Of note, a similar case was previously seen in a study of E. coli phage T4 and Lambda, where the receptor-binding tip of the T4 long tail fiber was found to be highly homologous to that of lambda side tail fibers (48). Thus, we postulated that baseplate-related genes could be transferred among viruses more frequently than genes for the major tail construction. Moreover, viral RBPs could evolve more rapidly than other baseplate-related counterparts. As aforementioned, R7M has undergone multiple horizontal exchanges at its baseplate, resulting a mosaic RBP composed of a RcGTA-like N terminus and a C terminus comprising a putative receptor interaction site targeting *Alteromonas*.

Hendrix et al. proposed that the vast majority of bacteriophages would undergo frequent horizontal exchanges of genetic elements from a global shared pool (49). Specifically, members of the *Nonagvirus* and *Seuratvirus* genera were reported to be isolated from animal fecal samples but also sewage samples, the *Nipunavirus* genus from sewage samples, and R7M and a few unassigned vibriophages from coastal seawaters. These reports highlight a possibility that domestic sewage discharge from inland to offshore might be a driving force of the evolution within the *Queuovirinae* subfamily. Moreover, these fast-growing hosts overlap their niches at times so that it is possible for their phages to communicate in a way of horizontal exchanges. On the whole, we hypothesize that these phages of a shared genetic pool should have a common ancestor and some of its offspring replaced their receptor interaction sites or entire baseplates through horizontal acquisitions from phages in the same environment, leading to the emergence of different host specificities. As a result, different evolutionary lineages that adapted to hosts of different taxa have evolved. A similar evolutionary scenario should also exist in viruses targeting slow-growing oligotrophs, and this could be supported by a recent finding in which three phages infecting the *Roseobacter* RCA lineage exhibited similar genomic content and architecture as SAR116 phage HMO-2011 except for tail adsorption apparatuses (50). The RCA and SAR116 strains are both well-known slow-growing yet dominant components in marine ecosystems. Future works of a larger scale to systematically investigate the existence of similar viral subfamily infecting copiotrophs or oligotrophs are needed.

Additionally, we reviewed two patterns of viral reproductive strategies observed here and also previous ones, as follows: (i) phages with a similar genetic framework may diversify their host spectrum through horizontal exchanges of their tail adsorption apparatuses, and (ii) phages with a different genetic framework can infect the same host through the lateral acquisition of similar tail adsorption apparatuses. The evolved phages have access to new hosts and phages of different genetic pools, which significantly promotes viral evolution, diversifies microbial gene repertoires, and further impacts host evolution and ecology through phage-mediated HGTs.

### Conclusions

This study isolated and fully characterized a siphophage, vB_AcoS-R7M, infecting *Alteromonas*. Along with the physiological characterization, genomic, structural proteomic, phylogenetic, and comparative genomic analysis have been conducted. R7M shows strong lysis ability to its host strain and is able to do interspecific infections within the genus *Alteromonas*. Numerous genes in R7M were found to be involved in phage-*Alteromonas* interactions, including genes involved in a host-independent UV-repair system, a putative AMG (*thyX*) that probably provides an alternative pathway for host nucleotide metabolism, and one class I AMG (*mazG*) and a few class II AMGs (*que*-related genes) hypothesized to promote viral propagation by regulating host metabolism. R7M was found to be evolutionarily relevant to a group of siphophages infecting diverse copiotrophs widely distributed in aquatic environments. Their similar features suggested that those phages shared a common ancestor, and we proposed the creation of a new subfamily (*Queuovirinae*) of *Siphoviridae* to group them. In-depth analyses of R7M revealed the coexistence of a RcGTA-like baseplate and a tail fiber with a putative receptor interaction site targeting *Alteromonas*. These results provide compelling evidence that the R7M distal tail has undergone multiple horizontal exchanges between diverse phage genomes. We hypothesize that horizontal exchanges in phage tail adsorption apparatuses could be a reason for the differential host specificities observed among the subfamily *Queuovirinae*. The replacement of viral distal tails (especially receptor interaction sites) is vital for phages to adapt to new hosts and new ecological niches. It also helps introduce new AMGs and phage-host interactions to host microbes. Overall, our comprehensive research of the alterophage R7M sheds light on the alterophage diversity, evolution, and ecology and deepens our current understanding of phage-*Alteromonas* interactions. Future works to explore the mechanism of transfer of tail adsorption apparatuses among diverse phages would help to exploit phage resources as a new genetic tool and give insights into phage therapy.

## MATERIALS AND METHODS

### Phage isolation and purification.

Alteromonas confluentis DSSK2-12^T^ was used as the bacterial host. It was grown in a rich organic (RO) medium (51) at 28°C with a shaking speed of 160 rpm/min. A total of 50 ml of seawater sample was collected from the coastal surface water on 14 October 2018, in Xiamen, China. A virus-containing sample was prepared by filtering the seawater sample through a 0.22-μm membrane (Labscale, Millipore, Massachusetts, USA) to remove large protozoa and bacteria, subsequently added into an early log-phase host culture, and coincubated for 24 h to increase the titer of putative infectious virions. The mixed culture was then passed through a filter to remove cell particles. The filtrate was diluted and mixed with fresh host cultures to allow the chance for phage plaque formation using the double-layer agar method (52). After purifying five times, a well-separated plaque was collected and stored in sterile storage media (SM) buffer (50 mM Tris-HCl, 0.1 M NaCl, and 8 mM MgSO_4_ [pH 7.5]) at 4°C for further use.

### Preparation of high-titer phage particles.

Phage lysates were centrifuged at 10,000 × *g* at 4°C for 20 min and further filtered through 0.22-μm membranes to remove cellular debris. The filtrate was then treated with polyethylene glycol 8000 (10% [wt/vol]) and kept at 4°C overnight to precipitate virions. The phage pellets were obtained through centrifugation (10,000 × *g*, 4°C, and 60 min) and resuspended in SM buffer. To further purify the virions, the phage suspension was laid on an equivalent volume of cesium chloride (1.5 g ml^−1^) and centrifuged at 200,000 × *g* at 4°C for 24 h using an Optima L-100 XP ultracentrifuge (Beckman Coulter, CA, USA). The visible phage band was extracted and further desalted using sterile SM buffer.

### Transmission electron microscopy.

The phage morphology was characterized by TEM. Briefly, 20 μl of the desalted phage solution was spotted on carbon-coated copper grids (200 mesh). After 30 min of adsorption in the dark, the phage sample was negatively stained with 1% phosphotungstic acid for 1 min, followed by air drying for 10 min. Phage images were captured using a JEM-2100 transmission electron microscopy (JEOL, Tokyo, Japan) at 80 kV. The size of phage particles was measured from at least five TEM images using the ImageJ software (53).

### Determination of the host range.

A spot assay was used in this study to determine the lysis profile of the purified phage (54). Specifically, 1 ml of the overnight bacterial culture was mixed with 5 ml of molten soft agar (0.5%), after which the mixture was immediately poured onto a solid agar plate (1.5%). After 10 min of air drying, 5 μl of 1:100 phage serial dilutions were spotted onto the host bacterial lawn. The agar plates were subsequently incubated at 28°C and then manually inspected after 24 and 48 h. The tested strains included a collection of 18 type strains of species of the genus *Alteromonas* and 3 *Vibrio* spp. (listed in Table S1).

### One-step growth curve.

One milliliter of early log-phase host culture was first exposed to phages at a multiplicity of infection of approximately 0.001, after which the mixture was immediately placed in the dark for 5 min to promote phage adsorption. The virions which were not adsorbed were removed by centrifugation, and cell pellets were washed and resuspended in 100 ml of fresh RO medium. The phage suspension was then incubated at 28°C with a shaking speed of 160 rpm/min. Every 15 min, viral abundance was determined using the double-layer agar method. The burst size was calculated as the ratio between the number of virions at the growth plateau and the initial number of the infected host cells (55).

### Phage DNA extraction and sequencing.

The purified virions were treated with protease K (100 mg/ml), sodium dodecyl sulfate (SDS) (10% [wt/vol]), and EDTA (0.5 mol/ml; pH 8.0) and kept at 55°C for 3 h for digestion. The digested sample was then purified using phenol-chloroform-isoamyl alcohol (25:24:1 [vol/vol]) and chloroform-isoamyl alcohol (24:1 [vol/vol]) to remove any impurities. The phage DNA from the supernatant was sequentially precipitated using isopropanol and stored at −20°C overnight. The precipitate was washed twice with 70% ethanol before air drying and finally dissolved in sterile Tris-EDTA (TE) buffer (10 mM Tris-HCl and 1 mM EDTA [pH 8.0]). The phage DNA was stored at −80°C before sequencing. Phage genomic DNA was sequenced using the Illumina HiSeq 4000 platform with a 150-bp paired-end DNA library. The filtered reads were then assembled *de novo* using Newbler assembler version 2.8 (56) to generate the final assembled sequence.

### Genome annotation and comparative genomic analysis.

Phage putative open reading frames (ORFs) were predicted using the combined results from Prodigal (version 2.6.3) (57), MetaGeneAnnotator (version 1.0-0) (58), and the online GeneMarkS server (59). ORFs were further annotated by BLASTP, the NCBI conserved domains database (60), and Virfam (61), with a cutoff E value of <10^−3^. Putative tRNA genes were detected using tRNAscan-SE (62). A custom java script was used to draw viral gene maps. Pairwise comparisons of viral gene contents were performed using an all-to-all BLASTP, with a bit score of >40.

### Structural proteome.

A total of 50 μl of a CsCl-purified phage suspension was mixed with 100 μl of SDT lysis buffer (4% [wt/vol] SDS, 0.1 M dithiothreitol [DTT], and 100 mM Tris-HCl [pH 7.6]) and the mixture was treated with boiling water bath for 10 min and then separated by standard SDS-PAGE. The SDS-PAGE gel slice was then excised, trypsinized, and analyzed using electrospray ionization tandem mass spectrometry (ESI-MS/MS), as described elsewhere (63).

### Protein structure alignment.

Predicted 3-D models of desired proteins were generated using I-TASSER (64) and visualized using PyMOL (65). The program TM-align (44) was employed to align two protein structures, with a TM-score of >0.5 indicating the same topology. The secondary structure prediction of rcc00171 and R7M tail fiber (ORF41) was performed using Jpred4 (66). Structure and amino acid sequence alignment of ORF41 against rcc00171 and other tailed viruses was performed using HHpred (43) and BLASTP, respectively.

### Network analysis.

A total of 3,464 genomes (358,468 proteins) of prokaryotic viruses downloaded from NCBI Refseq (version 99) was calculated for similarity scores compared with R7M using vConTACT2 (45), which defines viral clusters (VCs) as well. For clarity, viruses showing similarity scores of <1 to R7M were excluded for subsequent analysis. A protein-sharing network was visualized using Cytoscape 3.8.0 (67). The edge-weighted spring-embedded model was selected with viral similarity scores as the weight so that viral genomes sharing more viral protein clusters (PCs) will be arranged more closely together.

### Phylogenetic analysis.

To provide an overview of R7M lysis profiles, 16S rRNA gene sequences of type strains of species of the genus *Alteromonas* were downloaded from the NCBI database and used for constructing a neighbor-joining tree in MEGA 7 (68) with 1,000 bootstraps.

To understand phage phylogenetic relationships, complete amino acid profiles of all known alterophages and phages predicted to interact with R7M (vConTACT2 similarity score, >1) as well as their close relatives of the same genus were submitted to the VICTOR server (https://ggdc.dsmz.de/victor.php) for the tree building. All pairwise comparisons of viral amino acid sequences were performed using the genome BLAST distance phylogeny (GBDP) method (69) under settings recommended for prokaryotic viruses (70). The resulting tree was rooted at the midpoint with branch support inferred from 100 pseudobootstrap replicates.

Moreover, amino acid sequences of the major capsid protein, terminase large subunit (TerL), and DNA polymerase B (PolB) were used for marker gene phylogenies. Homologs were retrieved from corresponding viral PCs built by vConTACT2 (45). Maximum-likelihood phylogenies were constructed using IQ-TREE (version 1.6.12) (71) with best-fit models (VT+I+G4 for the capsid tree; LG+F+I+G4 for TerL and PolB trees) and 1,000 bootstraps. The four hallmark RcGTA-like baseplate-related genes that are mostly preserved in viral genomes were also included in phylogenetic analyses to explore the evolution of R7M distal tail components. Homologs were retrieved from the NCBI nonredundant (nr) database using protein sequences as the queries and subjected to a maximum likelihood tree in MEGA 7 (68) with 1,000 bootstraps.

Finally, all phylogenetic trees present in this study were visualized and manipulated using iTOL v6 (72).

### Data availability.

The complete phage genome of R7M has been deposited in the GenBank database under the accession number MT345684.1.
