# Supplementary material for: A Novel Phage Infecting Alteromonas Represents a Distinct Group of Siphophages Infecting Diverse Aquatic Copiotrophs
Source: mSphere. 2021 Jun 9;6(3):e00454-21. doi: 10.1128/mSphere.00454-21 (PMC8265664; doi:10.1128/mSphere.00454-21)
Supplement: TABLE S1 [file msphere.00454-21-st001.pdf]

TABLE S1 Strains used in the host-range test and their susceptibility to R7M.

Bacterial species	Strain	Origin	R7M
Alteromonas confluentis	DSSK2-12^T	Junction between ocean and a freshwater spring in Korea	+
Alteromonas hispanica	F-32^T	Hypersaline water from Fuente de Piedra, Spain	+
Alteromonas naphthalenivorans	SN2^T	A tidal flat in Korea	+
Alteromonas stellipolaris	ANT69a^T	Antarctic sea	+
Alteromonas addita	R10SW13^T	Seawater from the Chazhma Bay, Sea of Japan	+
Alteromonas litorea	TF-22^T	Intertidal sediments of the Yellow Sea in Korea	-
Alteromonas simiduii	AS1^T	Er-Jen River estuary	-
Alteromonas mediterranea	DE^T	Urania Basin, Adriatic Sea	-
Alteromonas aestuariivivens	JDTF-113^T	Sediments from a tidal flat in Korea	-
Alteromonas macleodii	ATCC 27126^T	Superficial waters in Oahu, Hawaii, Pacific Ocean	-
Alteromonas marina	SW-47^T	Seawater of Hwajinpo beach in the East Sea in Korea	-
Alteromonas tagae	AT1^T	Er-Jen River estuary	-
Alteromonas genovensis	LMG24078^T	Marine electroactive biofilm, Italy	-
Alteromonas australica	H17^T	Seawater from St Kilda Beach, Tasman Sea	-
Alteromonas gracilis	9a2^T	Sediments in the Pacific Ocean	-
Alteromonas lipolytica	JW12^T	Surface seawater of the Arabian Sea	-
Alteromonas pelagimontana	5.12^T	Sediments from the Southwest Indian Ridge, Indian Ocean	-
Alteromonas alba	190^T	Seawater from the West Pacific Ocean	-
Vibrio parahaemolyticus	ATCC 17802^T	Outbreak of “Shirasu” food poisoning, Japan	-
Vibrio campbellii	JL3507	Shallow-sea hydrothermal system in the Kueishantao Islet	-
Vibrio owensii	JL2581	Surface seawater of the South China Sea	-

^{T}type strains of species; +, sensitive; -, insensitive
