# Supplementary material for: A Novel Phage Infecting Alteromonas Represents a Distinct Group of Siphophages Infecting Diverse Aquatic Copiotrophs
Source: mSphere. 2021 Jun 9;6(3):e00454-21. doi: 10.1128/mSphere.00454-21 (PMC8265664; doi:10.1128/mSphere.00454-21)
Supplement: TABLE S2 [file msphere.00454-21-st002.pdf]

TABLE S2 Mass spectrometry data for vB_AcoS-R7M. A minimal of two unique peptides and 5% sequence coverage was used as threshold values.

ORF	Putative function	Mol. weight [kDa]	No. of peptides	No. of unique peptides	Sequence coverage (%)	coverage (%)
23	Portal	54.853	33	33	71.1	5.75
24	Minor head	71.441	33	33	52.3	2.06
26	Major capsid protein	37.984	34	34	78.2	53.33
28	Hypothetical protein	125.37	40	40	41.2	12.85
29	Head completion adaptor	19.503	9	9	49.2	1.70
30	Head-closure protein	13.278	5	5	76.2	0.27
31	Tail component	15.08	4	4	40.7	0.05
32	Tail terminator protein	15.329	9	9	66.4	1.31
33	Major tail structural protein	34.137	15	15	61	10.58
36	Tail length tape measure	85.319	50	50	63.3	5.32
37	Distal tail	22.521	8	8	55.9	1.18
38	Tail hub	30.978	10	10	45.4	0.47
40	Megatron	170	56	56	48.6	3.74
41	Tail fiber	28.476	12	12	70.3	1.33
