# Supplementary material for: A Novel Phage Infecting Alteromonas Represents a Distinct Group of Siphophages Infecting Diverse Aquatic Copiotrophs
Source: mSphere. 2021 Jun 9;6(3):e00454-21. doi: 10.1128/mSphere.00454-21 (PMC8265664; doi:10.1128/mSphere.00454-21)
Supplement: FIG S1 [file msphere.00454-21-sf001.pdf]

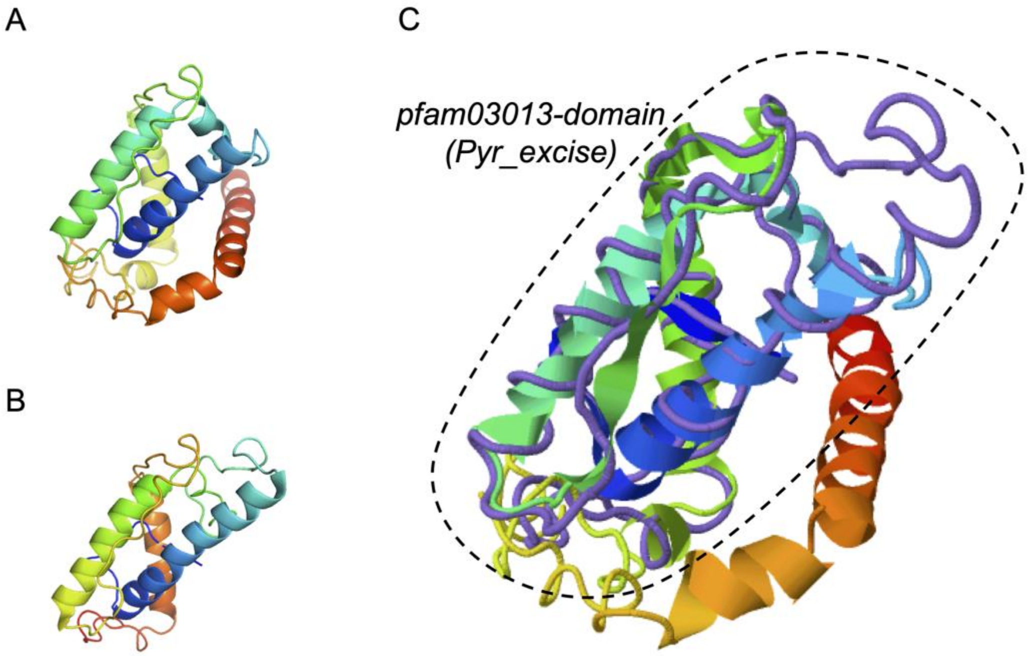

Supplementary References

1. Morikawa K, Ariyoshi M, Vassylyev DG, Matsumoto O, Katayanagi K, Ohtsuka E. 1995. Crystal structure of a pyrimidine dimer-specific excision repair enzyme from bacteriophage T4: refinement at 1.45 and X-ray analysis of the three active site mutants. J Mol Biol 249:360-375.
