# Supplementary figures and images for: A Novel Phage Infecting Alteromonas Represents a Distinct Group of Siphophages Infecting Diverse Aquatic Copiotrophs
Source: mSphere. 2021 Jun 9;6(3):e00454-21. doi: 10.1128/mSphere.00454-21 (PMC8265664; doi:10.1128/mSphere.00454-21)

A

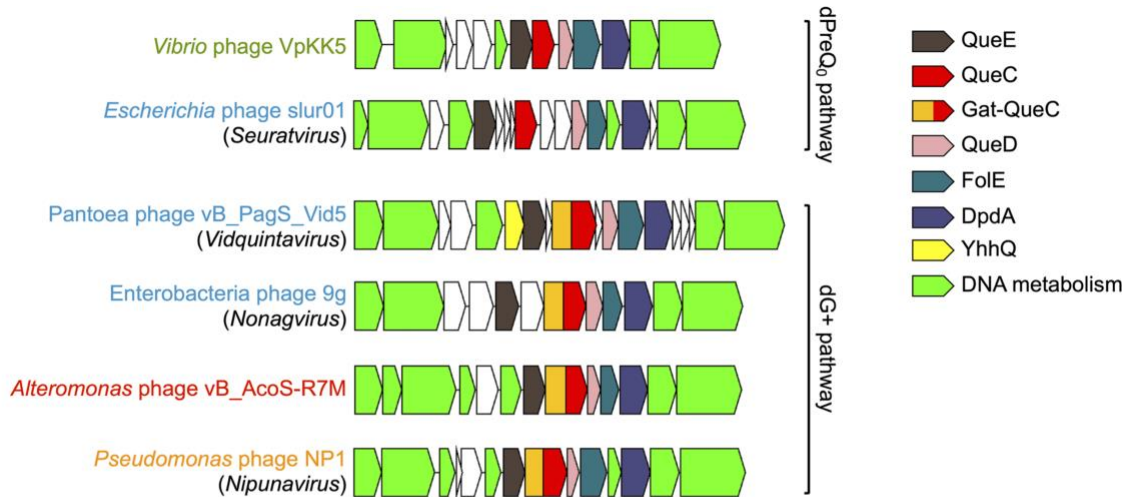

B

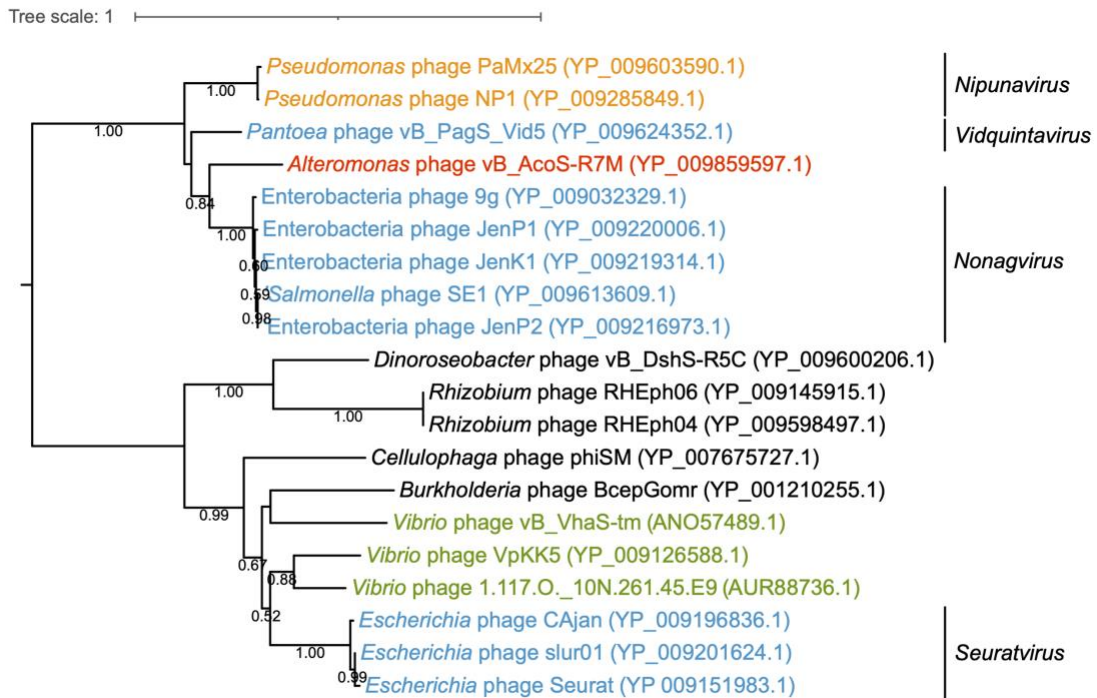

Supplement: FIG S2 [file msphere.00454-21-sf002.pdf]

A

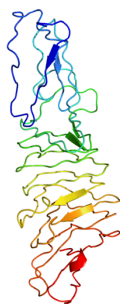

B

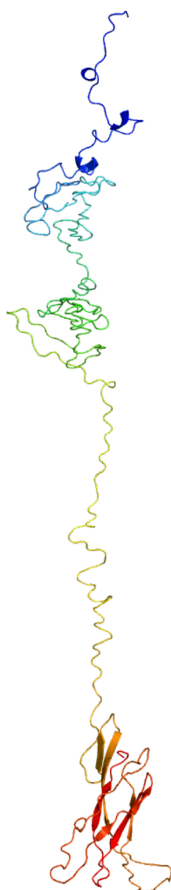

C

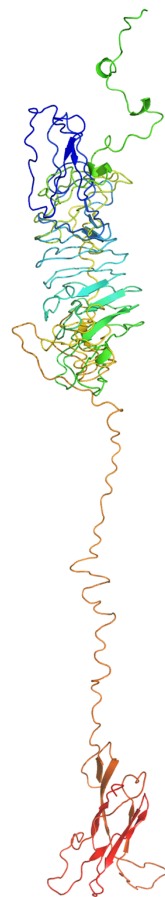

Supplement: FIG S3 [file msphere.00454-21-sf003.pdf]

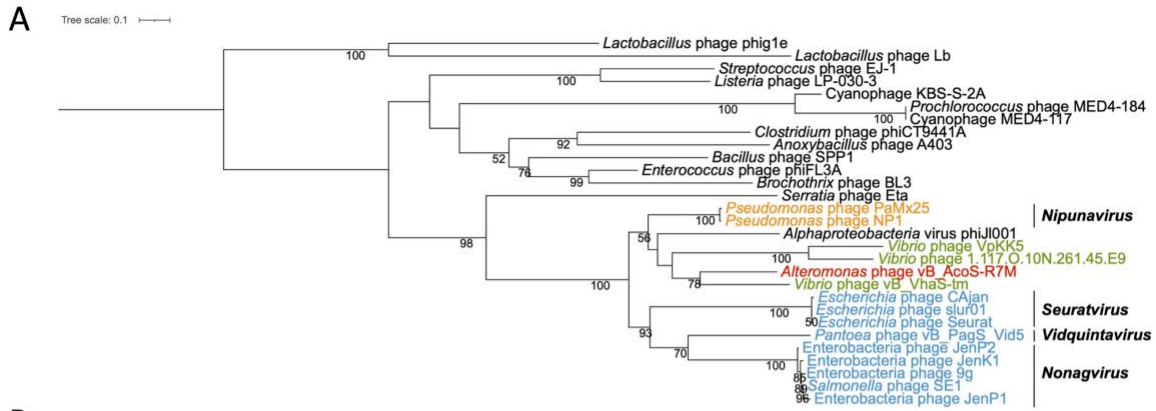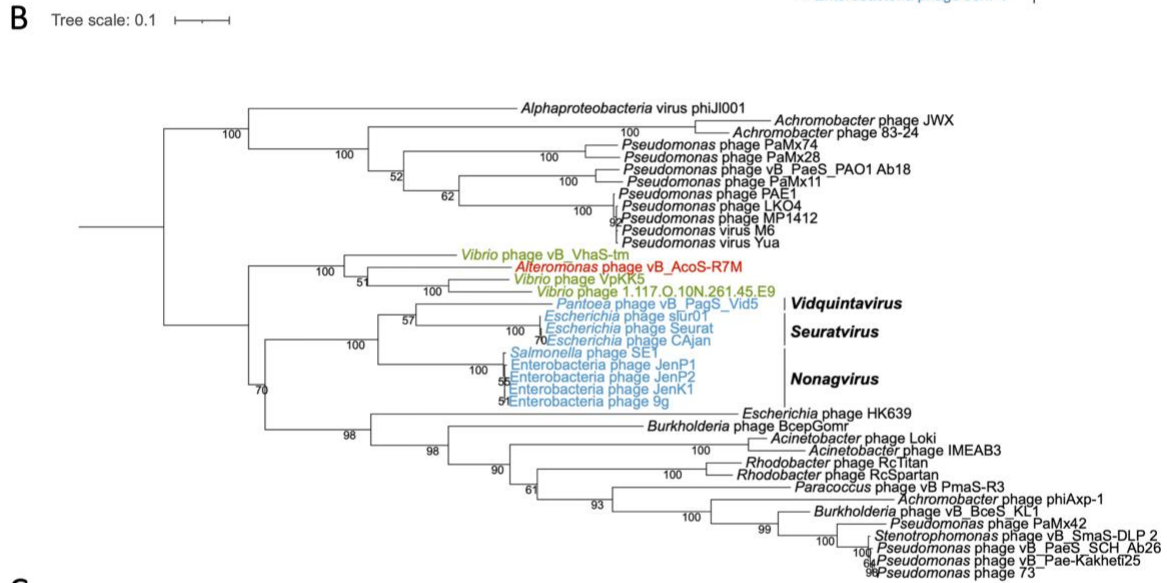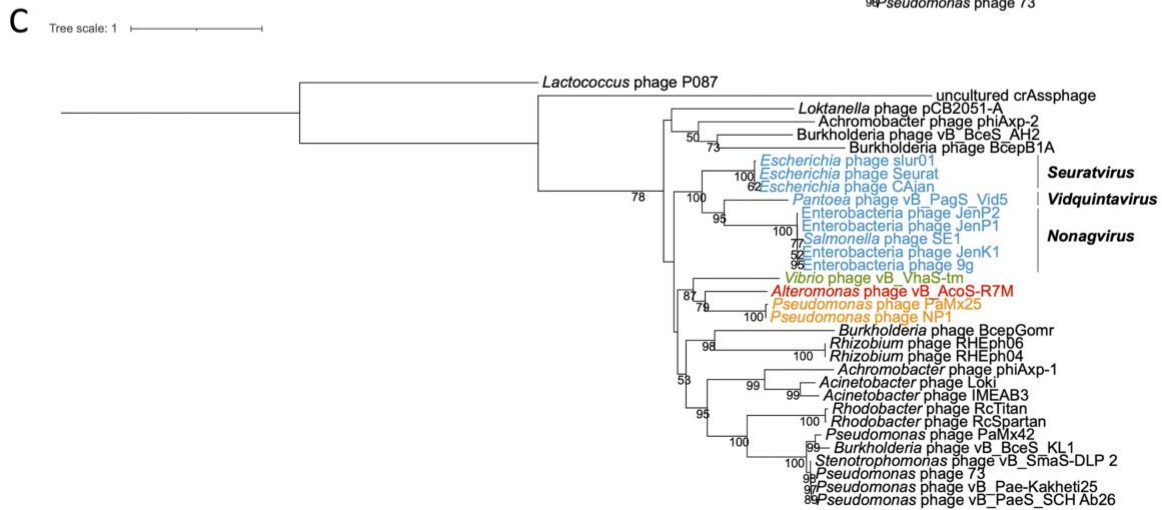

Supplement: FIG S4 [file msphere.00454-21-sf004.pdf]
